# A Proposal for a Data-Driven Approach to the Influence of Music on Heart Dynamics

**DOI:** 10.3389/fcvm.2021.699145

**Published:** 2021-08-20

**Authors:** Ennio Idrobo-Ávila, Humberto Loaiza-Correa, Flavio Muñoz-Bolaños, Leon van Noorden, Rubiel Vargas-Cañas

**Affiliations:** ^1^Escuela de Ingeniería Eléctrica y Electrónica, PSI - Percepción y Sistemas Inteligentes, Universidad del Valle, Cali, Colombia; ^2^Departamento de Ciencias Fisiológicas, CIFIEX - Ciencias Fisiológicas Experimentales, Universidad del Cauca, Popayán, Colombia; ^3^Department of Art, Music, and Theatre Sciences, IPEM—Institute for Systematic Musicology, Ghent University, Ghent, Belgium; ^4^Departamento de Física, SIDICO - Sistemas Dinámicos, Instrumentación y Control, Universidad del Cauca, Popayán, Colombia

**Keywords:** noise, music, machine learning, deep learning, performance evaluation, research methodology

## Abstract

Electrocardiographic signals (ECG) and heart rate viability measurements (HRV) provide information in a range of specialist fields, extending to musical perception. The ECG signal records heart electrical activity, while HRV reflects the state or condition of the autonomic nervous system. HRV has been studied as a marker of diverse psychological and physical diseases including coronary heart disease, myocardial infarction, and stroke. HRV has also been used to observe the effects of medicines, the impact of exercise and the analysis of emotional responses and evaluation of effects of various quantifiable elements of sound and music on the human body. Variations in blood pressure, levels of stress or anxiety, subjective sensations and even changes in emotions constitute multiple aspects that may well-react or respond to musical stimuli. Although both ECG and HRV continue to feature extensively in research in health and perception, methodologies vary substantially. This makes it difficult to compare studies, with researchers making recommendations to improve experiment planning and the analysis and reporting of data. The present work provides a methodological framework to examine the effect of sound on ECG and HRV with the aim of associating musical structures and noise to the signals by means of artificial intelligence (AI); it first presents a way to select experimental study subjects in light of the research aims and then offers possibilities for selecting and producing suitable sound stimuli; once sounds have been selected, a guide is proposed for optimal experimental design. Finally, a framework is introduced for analysis of data and signals, based on both conventional as well as data-driven AI tools. AI is able to study big data at a single stroke, can be applied to different types of data, and is capable of generalisation and so is considered the main tool in the analysis.

## Introduction

While the electrocardiogram (ECG) is an electrical recording of heart activity, heart rate variability (HRV) is a measurement derived from the ECG signal that provides information about the state or condition of the autonomic nervous system (ANS) (1). Given that HRV is linked to autonomic cardiac control, it has been studied as a marker for diverse diseases (2), both physical (3) and psychological (4)—e.g., for coronary heart disease, myocardial infarction, and stroke (5). HRV has been used to observe, among other indicators, the effects of medicines (6, 7), emotional responses (8), and the impact of exercise (9). Research has shown that HRV is affected by stress, as a result of low parasympathetic activity; stress reflects a sympathetic dominance (10), while relaxation states are associated with parasympathetic dominance (11, 12). Just as it has been measured to observe the effects of various elements on the human body, HRV has also been considered in assessing the effects of sound and music (13). Studies have been undertaken to look at the effect of music therapy (14) and diverse soundscape conditions (15). Given that it is a measurement related to both ANS and the state of the heart, HRV is a crucial record able to provide information about multiple aspects that might react to music. These include changes in stress levels (16), emotions (17), anxiety (18), subjective sensations (19), and blood pressure (13).

Although ECG and HRV are widely used in research related to health and perception, the studies vary greatly in their methodology. Koelsch and Jäncke therefore affirm the need to develop high-quality systematic research to study the effects of music on the heart—implying the implementation of standardised methodologies in this type of research (13, 20, 21). From experimental design to methods of data analysis and reports, huge variation is found. Researchers have therefore proposed several recommendations about measuring HRV for experiment planning, data analysis, and data reporting (22). Nevertheless, as the recommendations generally address an global focus, it becomes necessary to develop methodologies focused on specific stimuli such as sound and music and their characteristics (21). These methodologies should be guided towards how stimuli are able to affect physiological signals such as HRV (23) and autonomic control of the heart (24). Bearing in mind these conditions, this document proposes a methodological framework to design new experiments to study the effects of such sounds as musical structures and noise on ECG and HRV signals; the need for this framework arises as a result of the difficulty of making comparisons between many of the studies carried out on this topic.

The framework reveals just how complex any research examining the effects of sound on the heart might be. Shown within the framework are as many elements to be considered as possible, however clearly not all of these elements are presented in their entirety. Instead, providing general guidelines in this type of research and understanding that the topic is very complex, the framework makes no claim to be a set of unbreakable rules. Rather, the guidelines should be adapted to each study and the possibilities featured therein, the ultimate goal being that research on this topic might begin to be standardised. Future research could thus be more productive and even more conclusive as different studies will have a better chance of being able to be compared. Similar findings, as well as points of disagreement might then be illustrated, based on firmer foundations.

In the first instance, this document puts forward some elements of experimental design, such as definition of the experimental procedure, selection of subjects and sounds according to the research aims, and elements required for data collection. It then presents a framework for data analysis, in which it introduces the selection of data analysis techniques, methods of analysis of demographic, perception, ECG and HRV data, and elements to associate stimuli with ECG and HRV signals. Finally, some recommendations for the report of outcomes are presented.

## Framework Description/Details: Materials and Equipment

**Figure 1:**
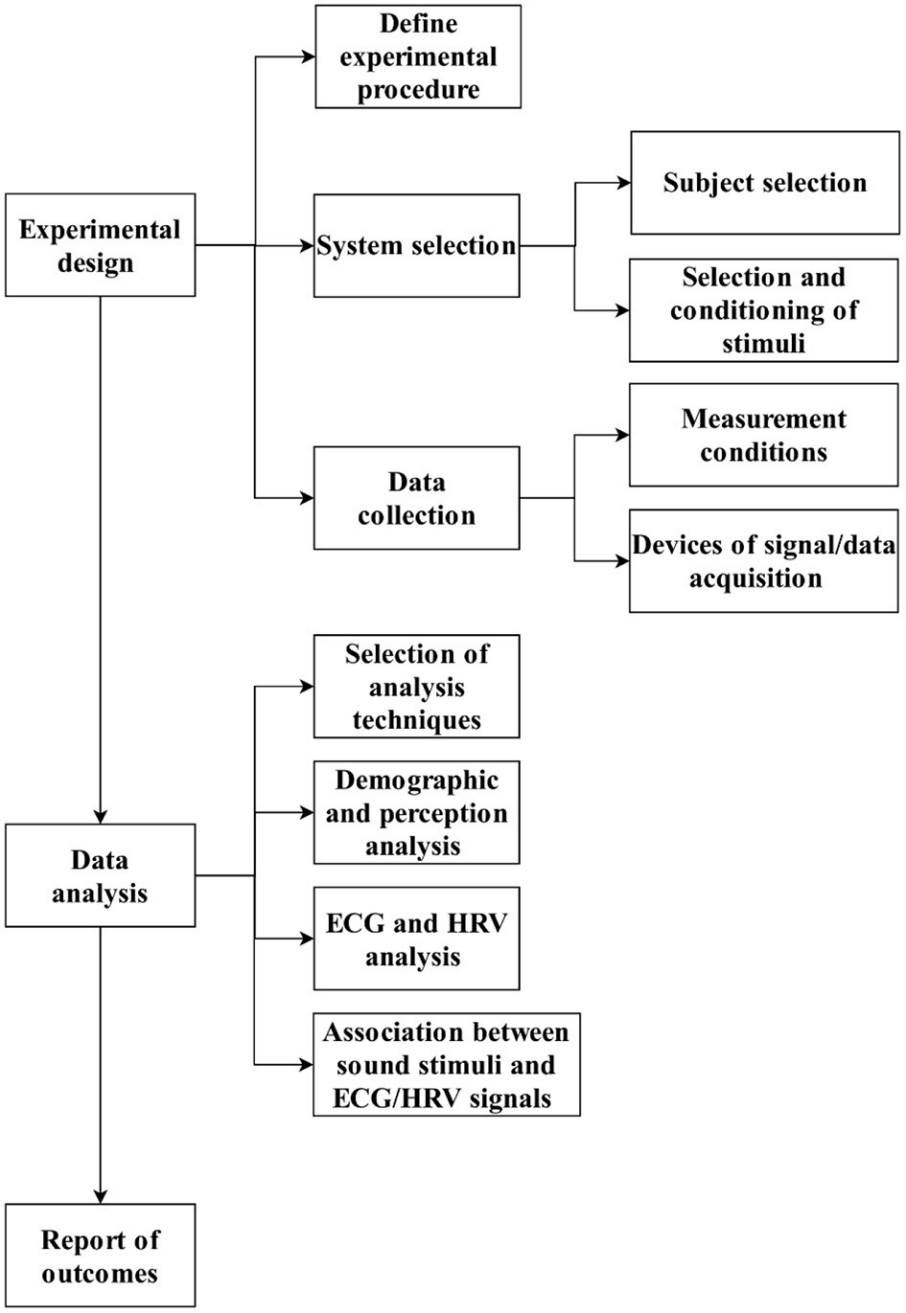
Block diagram of framework.

The framework comprises three main components (Figure 1): experimental design and procedure; data analysis; and report of outcomes. In the following sections, each is described along with the elements it contains.

## Experimental Design

The first phase involves experimental design. It is necessary here to clearly establish the aim of the study, according to which the experimental procedure will be defined, and subjects and sound stimuli selected. Devices for signal/data acquisition should be selected and measurement conditions optimised. Ideally, an interdisciplinary team should be formed to design the experiments, including health professionals (physicians, psychologists), experts in data analysis, computing science and statistics, and professionals in music or music therapy, with the resulting design benefiting from research from these disciplines. It is important to consider that, depending on the study aim, the experimental design ought to allow researchers to perform causality analysis, i.e., to establish cause-effect relationships.

### Defining Experimental Procedure

Definition of the experimental procedure is critical and involves carrying out as many pilots as necessary to fully adjust all procedures. Note that at this point, given the multiple design options, only general indications are provided.

#### Experimental Design and Procedure

In the design, it is necessary to select between existing experimental designs (25) or a mixture of several, taking account of advantages and disadvantages and the aim of the study (Table 1).

**Table 1:** Experimental designs and their description (25, 26).

**Experimental design**	**Description**
Between-subjects (27)	Each stimulus or set of stimuli is administered to a different group of subjects, minimising learning and transfer across conditions; shorter sessions, set-up more accessible than for within-subject.
Within-subjects (28)	All subjects are exposed to complete set of stimuli, one each time; fewer participants, minimising random noise.
Mixed (29)	Considers both between-subject and within-subject designs; allows evaluating effects of variables that cannot be manipulated in a within-subjects design because of irreversible carryover effects.
Nested (30)	Different possibilities exist in this category, including assignation of the whole group to the same stimuli, with different groups assigned to different stimuli; generality of outcomes is increased.
Combined (31)	This design includes more than one type of variable, for example combining correlational and experimental variables.
Correlational (32)	Descriptive method to determine if two or more variables covary and the nature of the relationships between them. Although less time-consuming than experimental research, the correlational method does not allow controlling observed variables nor establishing causality between them.
Pre-test–post-test (33)	This design performs pre-test of subjects on a dependent variable before presentation of stimuli; post-test is then carried out. Used to assess effects of change in observed variables; susceptible to carryover effects and requires control group.
Quasi-experimental (34) [Time Series/Samples (35), Interrupted Time Series]	Use quasi-independent rather than independent variables, observing the quasi-independent variable in naturally occurring conditions. No control over this variable should be carried out, however, such that use of a control group is suggested.
Developmental [cross-sectional (36), longitudinal (37), or cohort-sequential (38)]	Useful for figuring out changes in observable variables over time. Studies mostly focus on how age, cohort, gender, and social class influence development. In the cross-sectional approach, subjects of different ages are exposed to the same stimulus and studied at a unique time. In the longitudinal design, subjects are exposed to the stimuli several times during a specific time period. In the cohort-sequential approach, subjects from a cross-sectional sample are exposed to stimuli twice or more over a period of time.

Other relevant elements to consider in experimental design are physiological and psychological measurements, baseline measurement, stimuli presentation, possible carryover effects, and consideration of a control group. All physiological and psychological variables should be defined to achieve the research aim. Baseline measurement is recommended in all observable variables and may become a useful information source in the data analysis phase. The method of presentation of stimuli should be selected in such a way that study subjects will be as affected by them as much as possible. Experimental design should consider any source of carryover effects such as learning, fatigue, habituation, sensitisation, contrast, and adaptation (25). Design should reduce these unwanted effects. Finally, the design should establish consideration of inclusion or exclusion of a control group, based on the aim and hypothesis of the study, and conditions or limitations in resources and in the sample available.

It is good practise to consider a control group. The control group should not be in a state of silence and subjects in this group should receive a type of stimuli (13) different to that of the study group(s). Where a control is not possible, effects could be established in respect of subject baseline measurements.

### System Selection

#### Subject Selection

**Figure 2:**
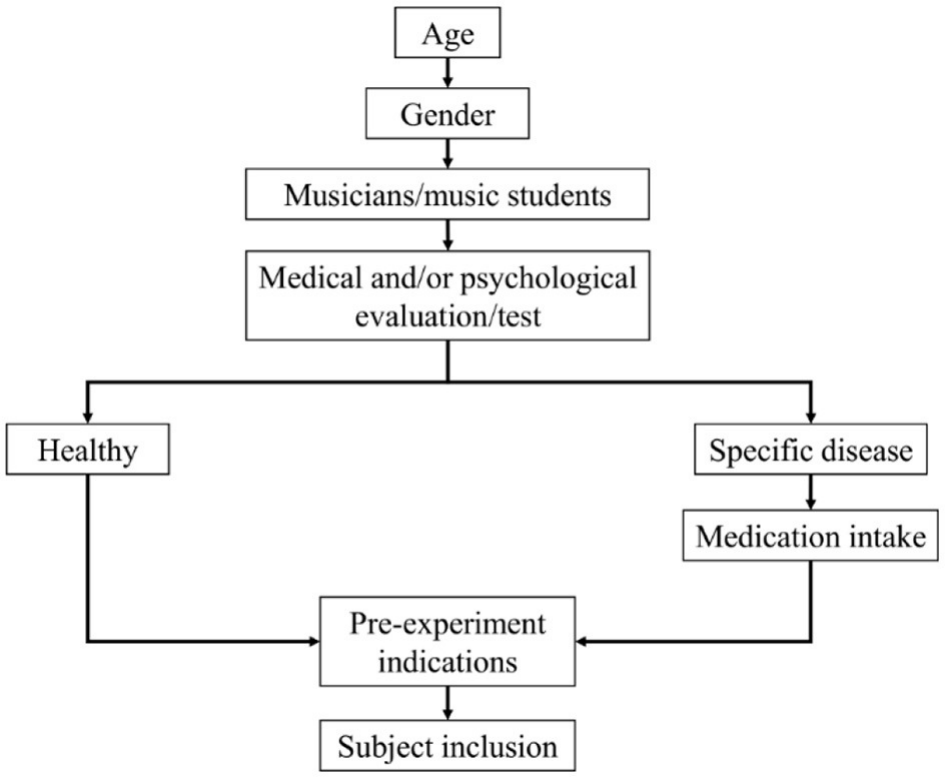
A general flow diagram to select and apply inclusion and exclusion criteria.

Given the study goal, the research population is selected (children, young people, men, women, with a particular disease or in good health) and inclusion and exclusion criteria (39) defined. A general flow process is proposed below (Figure 2):

Regardless of application of pre-determined inclusion and exclusion criteria, the following steps are proposed:

1. Selection of subjects according to age range and gender.
2. Definition of whether the research concerns musicians/music students or non-musicians, or a mix. Note that perception of music and noise in musicians could produce an outcome bias, as it may differ from that of non-musicians (40– 42).
3. Assessment of physical and psychological condition through medical evaluation or tests such as General Health Questionnaire (GHQ) (43), Medical Outcomes Study 36-item Short-Form Health Survey (SF-36) (44), Instrumental Activity of Daily Living (IADL) (45), Patient Health Questionnaire (PHQ) (46), and State-Trait Anxiety Inventory (STAI) (47). These evaluations can be applied in the inclusion and exclusion of subject; moreover, if performed after experimental procedures or as part of them, they could be considered as a source of information that might be susceptible to analysis as a response to stimuli or experimental procedures.
4. Selection of subjects based on their health conditions. According to the previous evaluation, healthy subjects or subjects with a specific disease should be chosen; in the latter case, it is essential to control the type of medicine taken, as some medicines could affect outcomes unexpectedly.
5. Providing subjects with all necessary indications before the experiments. Indications might include avoiding consumption of heavy meals, psychoactive substances, alcoholic beverages, stimulants, caffeine, and tobacco during at least 24 h before experiments or data acquisition. These substances could affect experimental measurements since they could produce unwanted effects both in physiological (heart rate, blood pressure) and psychological variables (anxiety) (48). Moreover, refraining from the practise of sport and exhaustive exercise is suggested between 24 and 48 h before data acquisition (49–51). Finally, on compliance with given instructions, subjects may be included for participation in the experimental phase.

#### Selection and Conditioning of Stimuli

**Figure 3:**
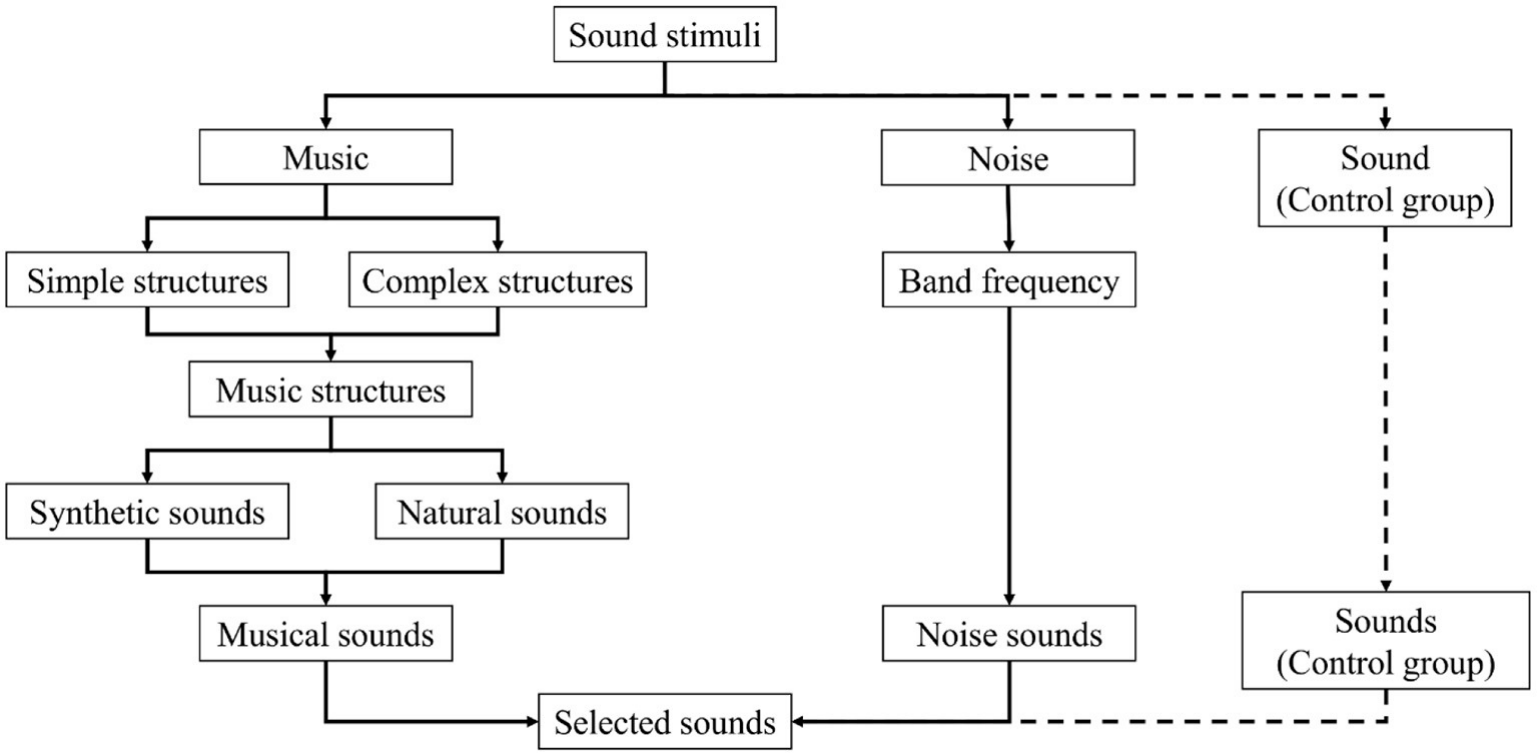
A general flow diagram to select the sound stimuli.

It should be decided if both music and noise are to be used. Nevertheless, where it is important to consider more than one type of stimuli, the use of an acoustical control stimulus is recommended (13). If the study is linked to music, music with lyrics and stimuli with verbal content should be avoided unless the experimental design requires it. Verbal content could produce additional effects (52), deviate outcomes or perhaps overcome effects produced by stimuli with no verbal content (52, 53). A general flow process is proposed to select sound stimuli (Figure 3).

Regarding the selection of music, it is first necessary to define if the research is to involve simple structures—scales, simple melodic lines, harmonic intervals, chords and simple progressions—for an in-depth analysis of the bare essence of music, or more complex structures—complex melodic lines, chord progressions, sections or complete songs or musical pieces—for a more general perspective. Having thus defined musical structures, it should be established whether these will be synthetic or natural. Where design permits, the use of synthetic sounds is recommended, since is possible to exert more control over several stimuli characteristics—frequency content (timbre), homogeneity in the range of sounds, and precision in playback. Synthetic sounds are also suggested if the study focuses on more profound or specific elements of music. In contrast, natural sounds might be preferred if the research has a more general approach.

Apropos of sound synthesis, it is possible to select between several methods according to sound type (54). For instance, to synthesise pitched sounds, it is recommended to use additive synthesis, concatenative/granular synthesis, frequency modulation synthesis, or oscillators (55). Alternatively, for acoustic instrument sounds, wavetables, waveguides/physical models, concatenative/granular synthesis, or additive synthesis (55) are encouraged. For textures and soundscapes, concatenative/granular synthesis, linear predictive coding, stochastic and wavelet-based methods (55) are favoured.

Concerning noise selection, noise sounds can be chosen principally by considering their frequency content, selected from six main types (56): brown, pink, white, grey, blue, and purple. Selection is made using frequency bands. For instance, to study low frequencies, pink or brown noise should be selected; for higher frequencies, blue or purple noise, while white or grey noise is suitable when considering all of the frequencies. Once sounds are selected, stimuli are presented in at least standard CD quality, i.e., sample rate of 44.1 or 48 KHz and 16 bits per sample (57). Similarly, synthetic sounds should meet sound/acoustic research standards, such as ISO 226: Acoustics—Normal equal-loudness-level contours (58); ISO 16: Acoustics—Standard tuning frequency (Standard musical pitch) (59); and ISO 266: Acoustics—Preferred frequencies (60). If there is no focus on effects of sound level, volume, intensity, or loudness, the loudness of all stimuli should be normalised, applying methods such as ReplayGain (61); the sound equivalent level in dB should be the same for all the considered stimuli. Furthermore, it is vital to consider sound levels according to the dose of sound energy. Following the WHO-ITU standard: Safe listening devices and systems (62) is advised.

### Data Collection

#### Consent Form

All procedures ought to be conducted under the Helsinki Declaration, where it should be ensured that subjects will not be at risk. A consent form with a description of the research and experimental procedures must be approved by a competent ethical committee and the form presented to be voluntarily signed by each subject before beginning the experimental process.

#### Measurement Conditions

For data acquisition, it is important to ensure study subjects are as comfortable as possible. In this way, outcomes are much less likely to be influenced by tiredness or discomfort. Maintain light exposure and influence of external sounds as low as possible. Subjects should be in a comfortable posture, e.g., in a supine position or sitting in a comfortable chair, to be more focused on the proposed task(s). They might also reduce body movement, improving the process of physiological signal acquisition. It is important to emphasise that the posture of the subjects needs to be standardised (supine or sitting) since gravity influences the baroceptors, affecting the HRV and the analysis that could be carried out.

Elsewhere, subjects should be alone during experiments. Where procedures allow, one subject per room is preferable, to avoid dealing with conditions involving more subjects. Researchers themselves should stay out of the experiment room. Where monitoring the subjects is necessary, remote use of a video camera is suggested.

#### Physiological and Psychological Measurements

For physiological measurements, in addition to the variables ECG and HRV, it is recommended to maintain a record of other variables likely to change. The acquisition of several electroencephalographic (EEG) channels is invaluable, as well as electrodermal activity (EDA) or galvanic skin response (GSR) measurement. Measurements of blood pressure (BP), respiration, and photoplethysmography (PPG), likewise. Measurement of psychological and perception variables (13) is also recommended; the STAI test, and recording perception using scales of valence, arousal, and dominance; to measure emotion, non-verbal assessment is recommended—Geneva emotion wheel (63) or Self-assessment manikin (SAM) where subjects can evaluate valence, arousal, and dominance on a pictorial scale (64); while a questionnaire on musical preferences of subjects and their listening habits (13) is recommended.

#### Devices for Signal/Data Acquisition

Hardware implementation enables measurement of the variables of interest. Having defined the physiological variables to be acquired, the devices that obtain the best quality measurements should be selected. Devices should also be selected to ensure the safety of study subjects and all personnel involved. All hardware devices should therefore comply with or be based on elements that comply with standards such as International Standardisation Organisation (ISO) or International Electrotechnical Commission (IEC). Devices or components should thus be made based on IEC 60601 for Medical electrical equipment: General requirements for basic safety and essential performance (65, 66); and ISO 13485: Quality management for medical devices (67).

Having ensured the safety of the subjects, the minimum sample acquisition rate (SAR) is determined, taking into account the frequency content of the physiological variables to be measured. According to the Nyquist principle, a sampling rate is required of at least twice the highest frequency of the signals to be recorded (68). An acquisition system with a minimum sample rate is therefore recommended based on the bandwidth of the signals (69): 250 Hz for ECG; 200 Hz for EEG; 10–100 Hz for EDA (70); 100 Hz for BP; 25 Hz for respiratory movements; and 1 Hz for PPG.

A final consideration regarding hardware implementation relates to standardisation of the sample rate. If the study requires a multisignal acquisition, all signals should be acquired with the same sample rate. In the event that signals are acquired with different sample rates, then in order to perform comparative analysis all of them must be resampled at the same sample rate. Finally, the acquisition system needs to be synchronised with the presentation of stimuli in such a way that it is possible to know the concordance between the presented stimuli and the acquired signals.

### Presentation of Stimuli

A double-blind study design is recommended (13), in which sounds should be presented using headphones, preferably with noise-cancelling in order to reduce possible effects of external sounds. Another possibility is the consideration of a triple-blind design, in which a blind statistical analysis might be applied to complement an AI analysis. It is suggested to consider stimulus duration of at least 10 s; this interval of time is recommended since it allows the establishment of a pattern behaviour in some aspects of the HRV analysis (22, 71). The inclusion of a period of silence between successive stimuli is suggested, to reduce a carry-over effect from the previous stimulus. The duration of this period of silence should be set, considering the general experimental design: the total duration of the experiment should be as short as possible while enabling the stated objective to be achieved. Shorter experiments are preferred to lengthier ones to reduce fatigue in the study subjects, minimising the impact of tiredness in the latter part of the experimental phases. Where possible, it is advised that study subjects keep their eyes closed as they listen to stimuli during the experiment; the use of a mask to cover the eyes would assist in avoiding the influence of visual stimuli on the measurements. This would also favour the capture of electroencephalographic signals as eyelid movements are reduced, minimising a source of noise.

### Baseline Measurement

Once informed consent has been signed, and before beginning the experimental procedures, a period of at least 15 min is suggested to allow all physiological variables to stabilise. After this period, a record of physiological variables should be performed to establish the baseline. Baseline measurements must be carried out under the same conditions as the rest of the experiment in order to compare outcomes. The baseline is used to determine if there was an influence of stimuli, in relation to it.

## Data Analysis

This section is focused on the more recent techniques of data analysis, specifically techniques of artificial intelligence: machine and deep learning. These types of technique have shown significant potential in data analysis and modelling. They are particularly useful and present advantages in comparison with traditional techniques when large time series with many variables are considered. Their use is encouraged since they can deal with a significant number of features. As shown in this paper, research on this topic has several sources of features or data; features can be extracted from signals such as ECG, HRV, and other physiological data. Moreover, data from perception, psychological variables and demographic data might be available. Analysis of sound stimuli would provide more features; with music stimuli, among other features it is possible to take account of pitch, tempo, loudness, and melodic and harmonic relations. Finally, the analysis of different subjects according to age or gender groups should be undertaken carefully during all stages of data analysis since it is important to bear in mind the physiological differences between them.

### Selection of Analysis Techniques

It is mostly necessary to use some kind of software to implement the experimental procedure (Table 2), to implement psychological tests or questionnaires with demographic information as well as to get information about listening habits or musical preferences. Additionally, software could be used to control the flow of actions in the experimental process—to implement an audio player with selected stimuli while at the same time presenting a graphical interface with which to obtain the perception scores of subjects.

**Table 2:** Software to analyse data and implement experiments in the behavioural sciences.

**Category**	**Name**	**Brief description**
Language, platform, or software	Matlab (72)	Multi-paradigm numerical computing environment and licenced programming language; very useful for data analysis and an option for producing sounds.
	Octave (73)	A free option similar to Matlab, containing many of its tools.
	Labview (74)	A graphical programming environment able to develop data analysis applications, user interfaces, and hardware integration.
	Python (75)	A multi-paradigm programming language that allows several possibilities for processing data, interacting with hardware, producing sounds, designing interfaces, and controlling experiments.
Software or packages	Psychtoolbox (76)	A free toolbox for Matlab and Octave for research in vision and neuroscience, enables synthesising and controlling visual and auditory stimuli, interacting with the observer, and an interface with computer hardware.
	PsychoPy (77)	A free cross-platform Python package to carry out experiments and paradigms in behavioural sciences; able to run experiments online.
	OpenSesame (78) and Psynteract (79)	A free software based on Python to conduct research in psychology, neuroscience, and experimental economics. It has a user-friendly interface and can run experiments online.
	PEBL (80)	A free software based on C++ Free psychology software for creating and design experiments. PEBL includes more than 50 psychological testing paradigms.
	PsyScope (81)	A software to design and run psychological experiments; runs on Apple Macintosh. Enables control of several kinds of stimuli, such as movies and sounds.
Online tool	Testable (82)	This tool allows for implementing behavioural surveys and experiments. It is possible to use stimuli. It is commercial software but has a free version.
	Gorilla (83)	This tool has a graphical interface and coding is not necessary. It allows collecting behavioural data and payment is required for each respondent.
	PsyToolkit (84)	This is a free toolkit for carrying out cognitive-psychological experiments and surveys. It has several survey and experiment libraries.

### Demographic and Perception Data Analysis

Demographic data are related to measurements of the size and composition of a particular population. This data is usually made up of descriptive information, including gender, age, marital status, household composition, ethnic background, state of health, education and training, employment status, income, and urban and rural residence (85). It is advisable to complete this information with preferences related to music knowledge and the listening habits of study subjects (86). Equally, it could be complemented with information about the subjects' perception or reported emotion.

Commonly, demographic data could be classified as nominal, ordinal, interval, or ratio. Its analysis might be performed with a descriptive or inferential character. Estimation of percentages, measures of central tendency and dispersion could be computed to enable a descriptive study (85). Measures of central tendency could be determined through arithmetic, geometric and harmonic means, median, and mode; measures of dispersion could be performed by means of variance, standard deviation, and quantiles. The relationships between groups or population characteristics could be studied if an inferential approach is sought (85). These relationships could be studied with tools such as correlation and regression. A review of the book “Methods of Demographic Analysis” is recommended for a complete overview of demographic data analysis tools (85).

### ECG and HRV Analysis

This section of the text presents the general methods used to process ECG and HRV data. However, it does not include the analysis of other physiological signals since it is not within the scope of this document. If the study to be undertaken includes other biosignals, revision of some documents related to EEG (87), EDA (64, 88), PPG (89), and BP (90) is suggested. This section therefore presents especially information related to digital signal processing, since ECG and HRV are generally recorded as digital signals. Additionally, AI techniques such as machine and deep learning are described, since they are considered as the main analysis tools in this proposal.

#### Introduction to Methodology of Analysis Using AI

**- Digital signal processing**

A signal is a variable phenomenon that changes with time (though it may vary with another parameter, such as space) and can be measured (91). Signals are processed through different tools or methodologies, which include statistics and digital signal processing (DSP). DSP is an instrument composed of a set of numerous mathematical tools designed for extracting, enhancing, storing, and transmitting useful information from a signal (92).

**Figure 4:**
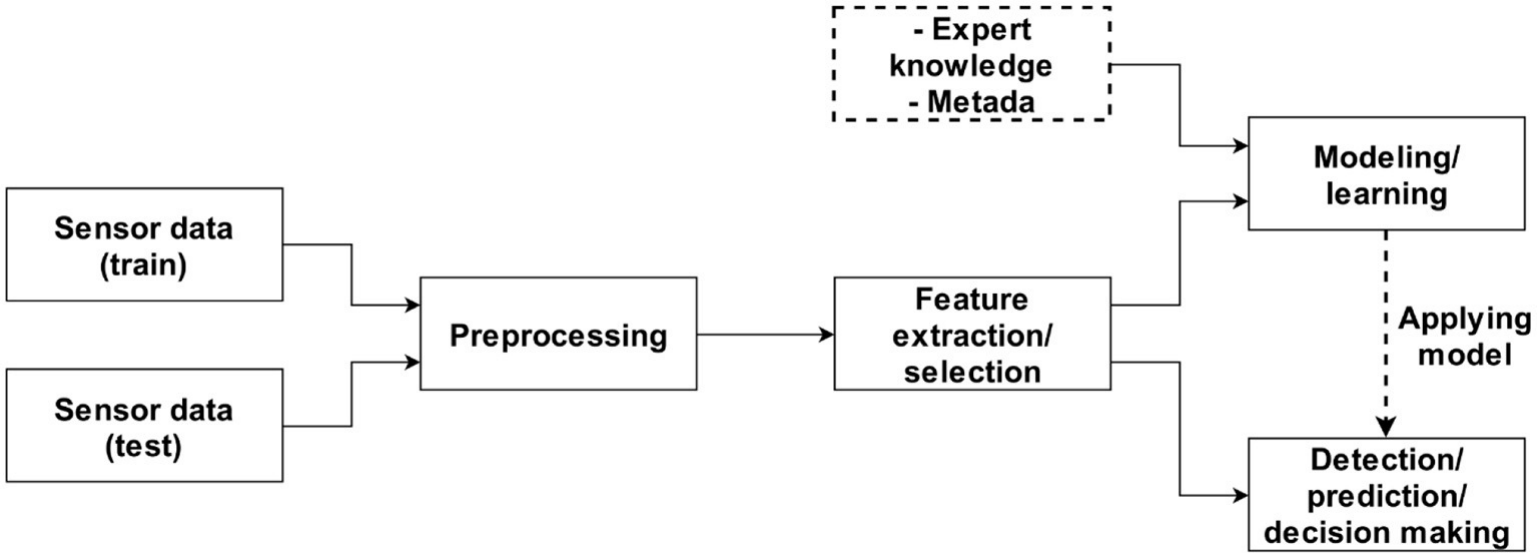
Stages of data and signal processing (93).

In many biomedical and bioengineering applications, data are collected using polls, standardised tests, medical or laboratory exams, and sensors. Once data is collected, it is analysed through different stages such as data pre-processing, feature extraction and selection, and data modelling (Figure 4) (93). In biomedical signal processing, pre-processing is commonly regarded as signal filtering; feature selection is also related to dimension reduction; and data modelling is associated with classification and, to a lesser extent, prediction. The modelling stage is also complemented by expert knowledge and metadata. These stages aim to perform the detection, prediction, and decision-making tasks, in which AI techniques such as machine and deep learning are used as part of digital signal analysis. In this regard, within the deep learning gamut, transfer learning is considered a very useful and powerful technique.

**- Artificial intelligence**

Artificial intelligence, or AI, is focused largely on tasks such as problem-solving and learning. For instance, a common application is the development of computational algorithms to distinguish between healthy and unhealthy subjects. AI seeks to develop algorithms to sort out real problems more efficiently than can humans (94) and has applications in several areas; however, in the context of this document the main implementations are related to expert systems, data mining or knowledge extraction, and knowledge representation (95).

Approaches based on AI present advantages compared to traditional analysis. AI might be used to study big data as a whole (96), it has the ability to “learn” features from a large volume of data (97), and it could also be utilised to study the influences of many features, initially, without having an idea which feature might be the most important one. AI also has the potential to study different types of data—features, time series, signals, and images. In general AI systems work without the need for human experts (expert systems: after training or development) (98). Other benefits of AI are related to efficiency, accuracy, and precision in analysis (99); competence to identify, classify and extract features from complex, high-dimensional and noisy data (100); the capability of generalisation; robustness; and the possibility to integrate expert knowledge (101).

#### Methodology of Analysis

##### Preprocessing: Signal Conditioning

**- Signal detrending**

Some non-stationary signals such as EEG, EMG, and HRV signals (102) are usually pre-processed in such a way that they could be considered as stationary signals. Detrending techniques are implemented so that the mean and standard deviation of the signals are almost constant (103). This procedure is widely used in treatment of signals such as EEG and ECG, and sometimes in HRV. Another way to deal with non-stationary signals is to analyse them by taking small parts in which they could be considered as stationary (103). Baseline wander is produced by different sources of low-frequency noise (104), among them a deficient contact of the electrodes to the skin and some effects of respiration as changes in impedance (105). Other techniques for detrending biosignals include cascaded moving average filter (105), quadratic variation reduction (104), wavelet transform (106), and adaptive filtering (107).

**- Signal filtering**

After removing baseline wander, a filtering stage is commonly performed to remove the noise of medium or high frequencies from several sources such as physiological variability, environmental noise or interference, transducer artifact, and electronic noise (103). A conventional filter stage is implemented to cancel interference from the power-line; in this case, it is possible to use anything from a notch filter with a cut-off frequency of 50 or 60 Hz to an adaptive filter (108). Concerning ECG signals, since ECG is affected by diverse noise sources (109, 110), the filtering process is a crucial stage that will influence systems analysis stages such as feature extraction and classification. Some techniques used are FIR and IIR filters (111), least mean square filters (112), wavelet transform (113), and Kalman filtering (114). Finally, after signal filtering, the signal to noise ratio (SNR) (115) is often used to assess the performance of the filtering stage.

**- R-peak/QRS-complex detection: HRV computation**

An important step following signal pre-processing is segmentation of R-peaks or QRS complexes. This segmentation is necessary in HRV computation since the analysis requires to know with precision the moment (occurrence) of each R-peak or QRS-complex (116). The element most used for detecting R-peaks is perhaps the Pan-Tompkins algorithm (117), other methods being linear regression algorithm (118), adaptive Hermite functions (119), adaptive bandpass filters and wavelet analysis (120), and CNN (121, 122). Once the R-peaks or QRS complexes are segmented, it is possible to compute the HRV signal. Thus, HRV is related to the variation between consecutive R-peaks, namely RR or NN intervals (116). HRV is normally presented as a time series expressed as the difference in milliseconds between successive R-peaks or QRS complexes.

##### ECG Feature Extraction

Some important information in ECG is represented by characteristics which are named features, and they are used for several purposes such as ECG filtering (123), ECG quality assessment (124), and disease classification (110). Many tools have been used to extract features from ECG signals, such as wavelet transform (113), PCA (125), statistics (126), analysis-based autocorrelation (127), Fourier transform (128), singular value decomposition SVD, variational mode decomposition VMD (129), Hilbert transform (130), and morphological methods (131). Several features have been extracted (132), for example, morphological features (commonly P, Q, R, S, T, and U waves) (121, 126, 133), statistical features (energy, mean, standard deviation, maximum, minimum, kurtosis and skewness) (126), wavelet features (coefficients and metrics extracted from continuous WT, Dual-Tree complex WT, tunable Q factor WT, flexible analytic WT and dyadic DWT) (134–136) and others, such as Lyapunov Exponents (137), the ratio of power spectrum (138), power spectral density (138), Kolmogorov Sinai entropy (137), and Kolmogorov complexity (137).

In addition to these elements, other tools could contribute to new approaches in ECG signal processing and analysis; e.g., fractal analysis. Thus, fractal geometry and multifractals allow analysing and processing complex shapes and signals (139). It is useful to look at the fractal or chaotic nature of ECG signals to inspect how the cardiac mechanism works, and so design modern approaches for analysis (140). More recent publications have shown that fractal analysis has contributed to the study of heart activity (141, 142). In addition to analysis with fractals, it is pertinent to bear in mind that this analysis can be used for the feature extraction process (143).

##### HRV Feature Extraction

HRV parameters are normally extracted from four different approaches—geometrical/non-linear analysis, fractal analysis, time domain, and frequency domain (Table 3) (150).

**Table 3:** Analysis of HRV signals.

**Analysis approach**	**Description**
Geometrical analysis	Geometric methods to analyse HRV allow transformation of RR intervals into geometric patterns (144). The most used geometric indices include Triangular index (TRI), a metric extracted from a histogram of normal RR intervals with all the RR values and their frequency of occurrence (5); triangular interpolation of normal RR interval histogram (TINN), a distribution of all RR intervals calculated from the base of a triangle shape formed from the histogram peaks (5); and the Poincaré or Lorenz plot, a graphical representation of HRV (145) obtained by plotting each RR interval as a function of the previous RR interval. From this plot, quantitative parameters are computed extracted from an ellipse fitted on the plot (144): length SD1 of a transverse line of that ellipse and length SD2 of its longitudinal line. Additionally, the SD1/SD2 ratio is taken into account (146).
Fractal and other analysis	Fractal analysis options include fractal dimension (147) and detrended fluctuation analysis (DFA) (148). Fractal dimension is often computed using the Higuchi and Katz algorithms (147), while in DFA two slopes α1 and α2 are produced, which describe brief and long-term fluctuations, respectively (148). Additionally, chaotic invariant and entropy analysis have been performed on HRV (147). Chaotic invariant analysis has used elements such as correlation dimension, largest Lyapunov exponent, and Hurst exponent (147, 148). The entropy approach has implemented tools such as Shannon spectral entropy, Kolmogorov Sinai entropy, approximate entropy, sample entropy, and Renyi's entropy (147, 148).
Time domain	Time indices of HRV are usually expressed in milliseconds. These metrics consider the mean and standard deviation (SDNN) of RR normal beats, the standard deviation of the means of RR (SDANN), the mean of the 5-min standard deviations of RR (SDNN), the root mean square of differences between adjacent normal RR (RMSSD, and the percentage of adjacent RR with a difference of duration >50 ms (pNN50) (149).
Frequency domain	Frequency-domain analysis commonly considers Fourier transform to extract ultra-low-frequency (ULF: 0–0.003 Hz), very-low-frequency (VLF: 0.0033–0.04 Hz), low-frequency (LF: 0.04–0.15 Hz), and high-frequency (HF: 0.15–0.4 Hz) bands (148). The LF/HF ratio is also usually considered.

**- HRV and time-frequency domain**

Frequently, continuous wavelet transform (CWT) is used as a time-frequency representation of ECG signals (151). However, this tool is not often used in analysing HRV (152); instead, Lomb-Scargle periodograms have occasionally been used (153). As such, this technique is proposed as an element to link HRV analysis to new techniques such as those related to deep learning. Several tasks could be performed through CWT by using algorithms such as convolutional neural networks.

##### Feature Selection—Dimension Reduction

After feature extraction, feature selection is required; here, several useful techniques have been used: wrappers (154) as a recursive feature elimination method (155), filters (156) as information gain (157, 158), and embedded, such as least absolute shrinkage and selection operator LASSO (159). It is sometimes necessary to reduce the number of selected features from the last stage by selecting the principal variables that best represent the signals and suppressing the ones with redundant information (dimensionality reduction). Moreover, PCA, LDA, Fuzzy C-mean, divergence analysis, ICA, and Fisher score are the most widely used dimension reduction tools in the applications with the best performance (160). In case of ECG analysis, among the most common tools are clustering methods such as *k*-means and hierarchical clustering (161), matrix factorisation methods such as singular value decomposition (SVD) (131), PCA (162), LDA (163), independent component analysis (ICA) (164), and other methods such as genetic algorithms (GAs) (164) and canonical correlation analysis (165).

##### ECG/HRV Modellin—Classification

**- Machine learning**

Classification is one of the final stages in analysing ECG signals. Most research develops systems for several tasks, such as disease classification (166), patient classification (167), ECG simulation (168), and emotion recognition (169). With this aim, supervised methods such as naive Bayes (170), random forest (171), genetic algorithms (128), linear and quadratic discriminants (172), SVM (173, 174), decision trees (175), discriminant analysis (138), and ANN (173, 174) have been used. In the same way, unsupervised methods such as hierarchical clustering (161), Gaussian mixture models (176), self-organising maps (177), and kNN (178) have been used. Modern methods of deep learning (179) such as CNN (180), long short-term memory (LSTM) (181), deep neural network (DNN) (182), robust deep dictionary learning (RDDL) (183) and restricted Boltzmann machine (RBM) (184) have been implemented.

Machine learning has contributed to various elements such as detection or classification of heartbeats (185), arrhythmias (129, 186), and unexpected changes in heart morphology (187, 188). Aspects related to the diagnosis of cardiac diseases and the analysis and classification of a considerable volume of ECG recordings have been improved. Real-time analysis and ECG simulations are growing topics (189). Regarding ECG classification techniques such as support vector machine, ANN, hidden Markov model, linear discriminant analysis, naive Bayes, and hybrid methods have been implemented (190). From the literature review, probabilistic neural network and support vector machine are observed as the classification algorithms with the most accuracy, higher than 98%, for cardiac arrhythmia detection (160).

Machine learning has been used to analyse ECG data (191), while these techniques have been used to a lesser degree to analyse HRV data. The extreme gradient boost (XGBoost) algorithm was thus implemented to find a connexion between HRV and long-term cardiovascular (192). Some algorithms such as logistic regression, support vector machine, random forest and AdaBoost were trained to classify between healthy and pulmonary patients from their HRV data (193). In addition to classification, XGBoost has been implemented to predict cardiovascular events from HRV parameters (194). Moreover, a Q-learning algorithm was implemented to associate HRV with the avoidance of negative emotional events (195).

**- Deep learning**

Among new machine learning methodologies, deep learning is being used in several areas related to biomedical engineering (196), perhaps due to the fact that deep learning performs better for large, diverse datasets than standard classification and analysis tools (179). Regarding ECG analysis, deep learning has been implemented in several applications (197), among them heartbeat classification, detection of coronary artery disease, myocardial infarction and congestive heart failure, detection and classification of arrhythmia, and detection and monitoring of atrial fibrillation (179). Analysis of ECG signals using deep learning has been implemented in applications related to sleep, to classify sleep stages and to detect obstructive sleep apnea (179, 196).

Even though deep learning is a new tool, published results have shown that it is possible to model several attributes of ECG signals and extract their particular features through deep learning (179). Convolutional neural networks (CNN) are the most used deep learning algorithms in analysis of several biological signals such as EEG, EMG, and ECG (179). One example is observed in Al Rahhal et al. (180), where the authors used CNN with continuous wavelet transform for detection and classification of arrhythmia. Another example is observed in (198), where deep CNN were used to detect atrial fibrillation.

Deep learning techniques have also been used to analyse HRV signals, though less often than for ECG signals. Deep neural networks were used to measure stress levels from HRV records (199). CNNs were used in emotion recognition tasks, where HRV was integrated with multiple physiological signals (200). An LSTM approach was implemented to identify sleep-wakes from acceleration data and HRV (201) and to detect congestive heart failure (202).

### Association Between Sound Stimuli and ECG/HRV Signals

**Figure 5:**
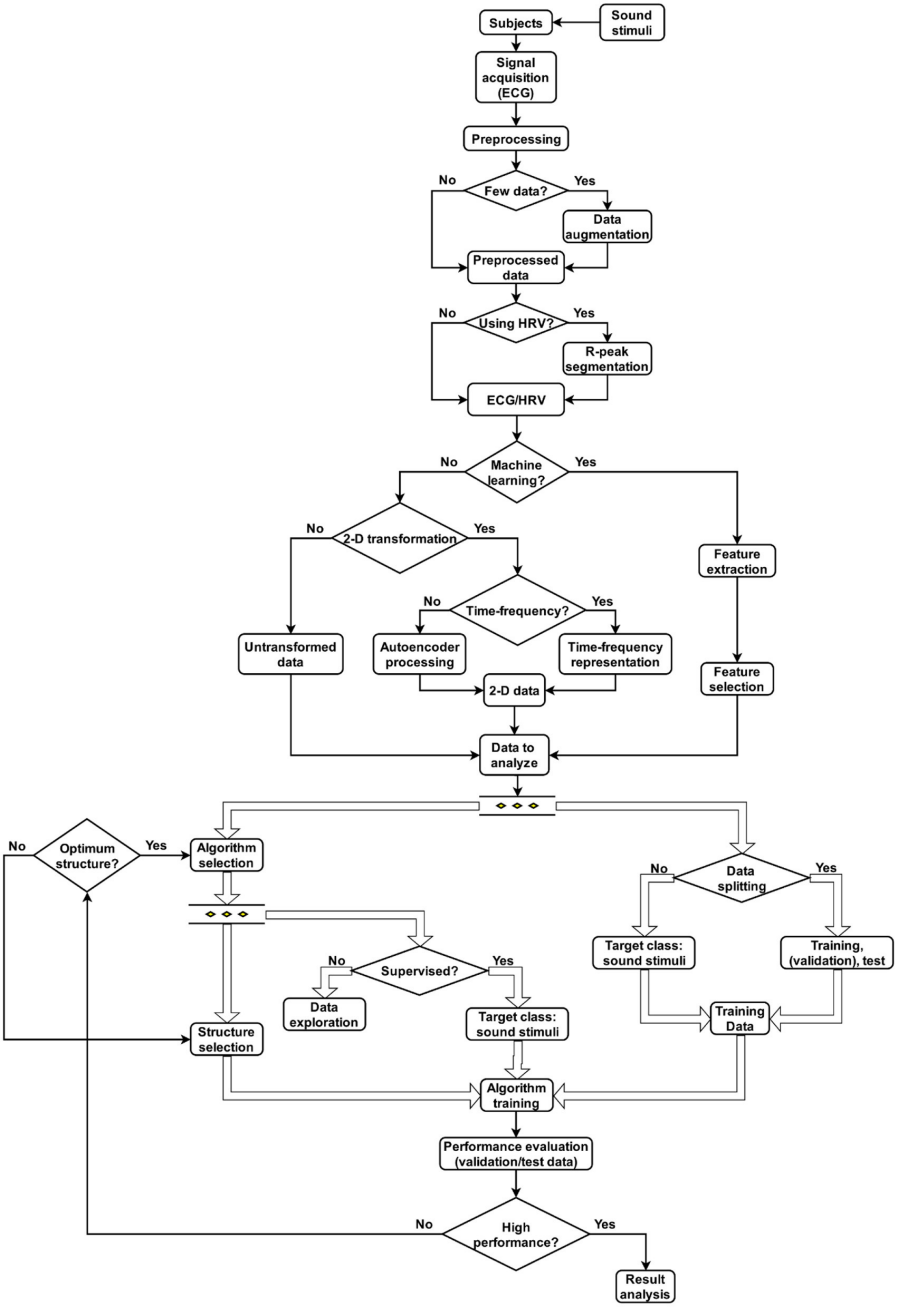
Methodology for the association between sound stimuli and ECG/HRV signals.

Having observed the advantages of using AI, and in particular the benefits that machine learning and deep learning bring to cardiovascular medicine (203) on including ECG and HRV analysis, it is clear these tools represent a valid method to associate these signals to sound stimuli. As such, it is proposed to consider classification tasks as an element of correlation between signal features, in a machine learning approach, or time-frequency representation of the signals, in a deep learning approach, and stimuli characteristics as target classes (Figure 5). In this approach, high performance in the classification process represents a good correlation between the signals measured and the stimuli presented. The association between stimuli and their effects on the heart should be carried out by means of HRV/ECG comparison between participants exposed to the same auditory stimulation.

Correspondingly, as described above, following signal acquisition, conditioning of the signal is necessary. Depending on the power source, applying a notch filter with a cut-off frequency at 50 or 60 Hz may well be required. In any event, some pre-processing techniques will also require to be applied (filtering techniques might be applied, for example, to remove the noise from different sources). The techniques should be chosen depending on the sources of noise present during the experiment as well as the acquisition system employed. Once signals have been suitably conditioned, if few data are found, a data augmentation process may be necessary. Augmentation may be required on applying machine learning techniques, whereas with deep learning techniques, it is almost always necessary. In contrast, the possibility exists of applying transfer learning techniques (204), where it is possible to deal with restricted data. Augmentation techniques suited to ECG signals might include noise addition (205), wavelet-based shrinkage filtering (206), and signal windowing or segmentation (206) with or without overlap. After signal pre-processing, if the research is focused on HRV, this should be extracted from the captured ECG signal. R-peaks must be segmented and subsequently, computing the time difference between successive peaks is required. With both ECG and HRV signals conditioned, the next phases of the study can begin.

At this point, the method for analysing the data is selected determined by the main aim of research and the amount of data available (machine or transfer learning if data are scarce; deep learning techniques where data is more abundant). If determining which data features or elements most affect outcomes is relevant (i.e., an exploratory, descriptive, or explanatory approach), machine learning techniques should be applied. Where the focus is on the results (i.e., without considering their provenance, in a relational, or applicative approach), transfer or deep learning tools apply. It is next necessary to prepare the data before applying it; if a machine learning technique was selected, some techniques of feature extraction from the data are likely required. After this procedure, where there is a large set of features, applying a feature selection method is recommended. If a transfer or deep learning technique was selected, applying a transformation of data into a two-dimensional representation—a time-frequency or autoencoder algorithm transformation—might be required (207).

The dataset should next be split into sets to train and test the selected algorithm; a validation set may be included. The training set should always be the larger set, usually 60–80% of total data. The remainder is assigned to the test set, or distributed between validation and test sets. The k-fold cross-validation method can reduce overfitting (208). As data is split into training sets, the algorithm to analyse data is chosen. If this algorithm is supervised, the target class could be established from different characteristics of the sound stimuli: an association between the training data and the selected characteristics could thus be performed. Following algorithm selection, the algorithm structure must be selected taking training data and research aims into account.

#### Performance Evaluation

It is then time to train the algorithm using the training set or k-fold cross-validation method. Performance evaluation with the test set follows, measured using such elements as the confusion matrix (209), and derived metrics such as F1 score, accuracy (208), and receiver operation characteristic (ROC) curve (210). Although these metrics are used widely, most have drawbacks (13). F1, for instance only takes into account positive classes, carrying bias by disregarding negative classes (13). Similarly, AUC does not take a classification threshold, which classifiers generally require (13).

Among other possible metrics, the Kappa coefficient (211) and Matthews correlation coefficient (MCC) have emerged strongly (212). By considering all elements of the confusion matrix, these provide a better idea of the general performance of machine learning algorithms (13, 213). The Kappa coefficient can take values between 0 and 1, for which Landis and Koch have proposed a scale for interpreting the coefficient (211), divided into intervals 0 to 0.20, 0.21 to 0.40, 0.41 to 0.60, 0.61 to 0.80, and 0.81 to 1.00 that indicate slight, fair, moderate, substantial, and almost perfect agreement, respectively. MCC is usually interpreted as a correlation coefficient because it can produce values between minus one and one (214). A value of one relates to a perfect classification; minus one indicates discrepancy between observations and prediction; and zero indicates an uncorrelated or random prediction (13). It must be noted that MCC can be used even with imbalanced datasets (214).

Given the framework aims, MCC is highly recommended for validating the performance of the selected machine learning tools. It can equally provide a quantitative measurement of the correlation between sound stimuli and the responses observed in the variables, since it can be interpreted as a correlation coefficient.

#### Metric for Applications Related to Harmonic Musical Intervals and Noise

In addition to using MCC, developing new metrics for each specific application is recommended (215, 216). One possibility involves implementing a cost matrix. In classification applications, the cost matrix provides information about the cost of wrong classifications. Cost is incremented with each instance incorrectly classified. This metric can consequently describe system performance and further be adapted to each specific application. Unlike most standard metrics, a cost matrix provides the fullest idea of right and wrong classifications.

If performance is evaluated as not sufficiently high, improvements are made to the system. Adjustments to the algorithm structure could be performed, or a different algorithm employed. Finally, when performance is high enough, data analysis can proceed. An example of application of this framework is again found in “Assessment of heart rate variability and heart response to harmonic music interval stimuli using a transfer learning approach.”

Building a cost matrix as evaluation metric is proposed, based on the following rules:

1. A hierarchical order must be established between classes to be classified, dependent on each specific case: e.g., in classifying harmonic musical intervals, a hierarchical order might be determined by degree of consonance or dissonance. The established order will allow assigning classification costs. Sorting classes into adjacent orders will cost less than sorting between more hierarchically distant classes.
2. When seeking to classify more than one general class, a hierarchy must be established for each general class: e.g., for two general classes A and B, with subclasses A1, A2, B1, and B2, two hierarchies are established, one for general class A and one for B. Maximum cost to pay is set equal to 1 (*C* = 1). This cost will be distributed among all possible classification options for each class. For more than one general class, a sub cost (SC) is defined for each class. This sub cost will be established dividing the maximum total cost by the total number of classes.
  $$SC=\frac{C}{\#GeneralClasses}$$
3. SC is then distributed among all subclass elements. If an element belonging to a subclass is classified within another subclass, a higher cost will be paid than if classified as an element of its same subclass. Taking the previous example, if an element in class A1 is classified within class A2, the cost to be paid will be less than if classified within any class of general class B, B1 or B2. In the case of the SC of different classes, this ought to be distributed equally among classes. Thus, in the case of classification of elements of general class A, classification of these subclasses as elements of subclass B will cost SC/2 where 2 corresponds to the number of classes, B1 and B2, of subclass B in this example.
4. For the SC of classes corresponding to the same subclass, the cost of correctly classified classes will have the minimum value, corresponding to zero. To distribute SC between erroneous classifications within the same subclass, three methods are proposed:
  $$\begin{array}{l} \text{ n}=\text{number of subclasses}\\ \text{SC}=\text{sub cost} \end{array}$$
  - Linear distribution: in this case a minimum cost is assigned, given by Equation 1, and the costs corresponding to the other classes according to Equation 2:
    (1) $$Cost_{Minimum}=\frac{SC}{\sum_{i=1}^{n-1}i}$$
    (2) $$SC_{i}=\{\begin{array}{c} 0,\;i=1\\ \left(i-1\right)*Cost_{Minimum},\;i=\left[2,n\right] \end{array}$$
  - Distribution based on the inverse-square law. Since various physical phenomena—gravity, electrostatics, and the radiation of light and sound—vary inversely to the square of the distance, allocation of costs is proposed taking this approach into account. Equation 3 is proposed to assign SC values:
    (3) $$SC_{i}=\frac{1-\frac{1}{i^{2}}}{\#GeneralClasses*\sum_{j=1}^{n}1-\frac{1}{j^{2}}},\;i=\;\left[1,n\right]$$
  - Distribution based on Gompertz function (217). As this function is widely used in the field of biology (description of growth of plants, animals, cells, bacteria, etc.), it is proposed as a tool for cost allocation. To assign SC values, Equation 4 is proposed:
    (4) $$\begin{array}{l} SC_{i}=\frac{e^{-\beta e^{-\gamma i}}-e^{-\beta e^{-\gamma }}}{\#GeneralClasses*\sum_{j=1}^{n}e^{-\beta e^{-\gamma i}}-e^{-\beta e^{-\gamma }}},\\ \;i=\;\left[1,n\right] \end{array}$$
    In this case β and γ represent constants that modify horizontal displacement of the function and growth rate, respectively.

Linear distribution is recommended where the cost of wrong classifications does not have great consequences, i.e. when the cost to pay for wrong classifications is not very high. This method could be used in classifying the same type of stimulus, but would be recommended in classifying heart disease and normal signals. Distribution based on the inverse square law is recommended for stricter applications, e.g. medical applications where a high cost is paid for wrong classifications. Finally, use of the Gompertz-based distribution is generally recommended. This function can be adapted according to each specific application, through constants β and γ and can be used for both flexible and strict cost applications.

Once established, the cost matrix is multiplied by the confusion matrix to determine the classification cost matrix. All values in the classification cost matrix are then added together to obtain the total cost. Finally, to standardise the value between 0 and 1, total cost is divided by maximum possible cost. Maximum possible cost is determined considering the case where each instance of the classes is classified as the class with the highest cost in the cost matrix.

#### Framework Validation

Following implementation, framework validation is a vital step (218), to determine whether it was well-adapted to the study in question and used to the full, or might be improved. Just as it was suggested above that an interdisciplinary team carry out the experiment design, so a multidisciplinary team is recommended for framework validation, which should be conducted from the viewpoint of each discipline involved in the research. The framework is evaluated according to application requirements and its implementation is then optimised under research conditions (219). Validation should confirm that the framework is within ethical considerations, all its procedures ensuring high quality scientific practise and should further assure that results can be compared with outcomes of other research performed under similar conditions. Such validation increases possibilities to obtain meaningful data. In this regard, some critical characteristics of testing and clinical validation presented in Beniczky and Ryvlin (220) were adapted to this framework as follows (Table 4):

**Table 4:** Stage of framework validation.

**Item**	**Stage**				
	**0**	**1**	**2**	**3**	**4**
**Subjects**					
Healthy subjects	+	+	±	±	±
Number of subjects	>10	>10	>20	>20	>20
**Recordings**					
Conventional methods	±	±	±	±	±
Dedicated device	+	+	+	+	+
**Analysis**					
Training & testing using the dataset	+	+	–	–	–
Predefined algorithm and cut-off values	–	–	±	±	±

*+, compulsory; ±, optional; –, excluded*.

**Figure 6:**
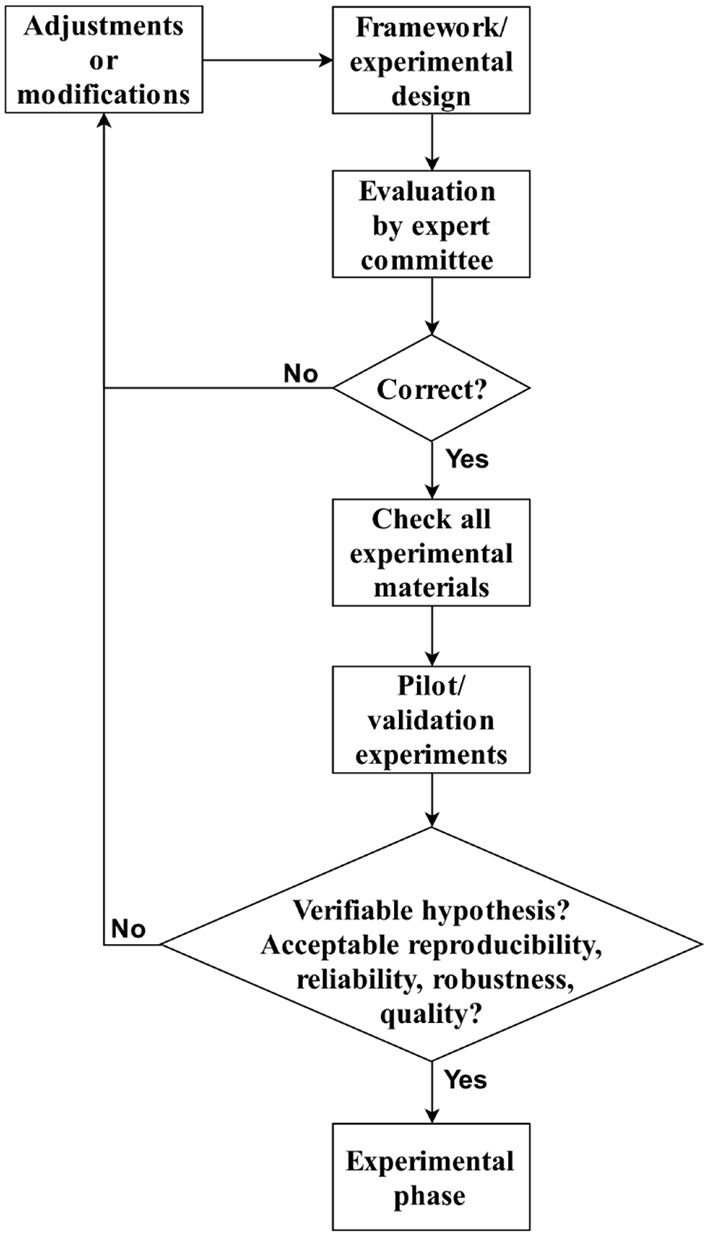
Framework validation protocol.

Validation should assess the quality, reliability, robustness, and consistency or reproducibility of the outcomes (221). As a validation protocol of this framework, carrying out the following steps is suggested (Figure 6):

1. Present the adapted framework to an expert committee.
2. Determine if the framework and the experimental design are correct. Otherwise, it is necessary to perform adjustments and go back to step 1.
3. Cheque if all experimental materials are working well: devices, stimuli, laboratory, or experimental place in optimal conditions.
4. Develop a set of pilots or validation experiments.

With the outcomes of the pilots, confirm if the hypothesis is verifiable and assess the quality, reliability, robustness, and consistency or reproducibility of outcomes. If this evaluation does not produce good results, it is necessary to perform adjustments and go back to step 1.

One method of verifying the validation protocol is by means of a checklist. Constructing a checklist of items included in the research is recommended (Table 5).

**Table 5:** Checklist of research elements.

**Research elements**	**Check**
Definition of experimental procedures	
Inclusion and exclusion criteria	
Health and music preference questionnaires	
Informed consent	
Available subjects	
Selection or generation of stimuli	
Devices of data acquisition	
Devices of stimulus presentation	
Review and adjustment of protocol—Pilot test	
Pilot test	
Data analysis	
Review and adjustment of protocol—Pilot test	
Beginning of the experimental phase	

This framework was inspired in the studies reported in the literature and also in our own research and experience in the fields of artificial intelligence, perception, music, and health. One example of the application of this framework can be observed in our most recent publication “Recognition of valence judgments in music perception using electrocardiographic signals and machine learning” (222)^1^.

## Some Suggestions to Present a Report of Outcomes

On completion of result analysis, it is essential to provide a report as a scientific paper regarding the findings of the research. Initially, it is necessary to make clear the objective of the study and the methodology with which to achieve it. Provide as much information as possible about the methodological procedure, describing the most critical aspects of the experimental procedure. Stimuli, method of presentation of stimuli, and physical conditions of the place of experimentation should also be described, together with the considered population and the methods employed to acquire data.

It is furthermore relevant to provide sufficient information about the methods used to analyse the data. Outcomes of the analysis should be presented clearly and precisely. Finally, it is essential to demonstrate the significance of the results and their relevance. A possible additional element is the presentation of limitations in the research and possible ways of improving similar research in the future. Wherever possible, it is a good idea to present new conceivable ideas to deal with new studies related to the research topic or the study aim. If it is desired to obtain a complete reference about research in music and its written reports, review of the book “Music in words: a guide to researching and writing about music” is recommended (223).

Other useful tools to bear in mind for reporting outcomes are: STROBE statement (STrengthening the Reporting of OBservational studies in Epidemiology) (224, 225); CONSORT statement (Consolidated Standards of Reporting Trials) (226, 227); SQUIRE stands (Standards for QUality Improvement Reporting Excellence) (228, 229); STARD initiative (Standards for Reporting of Diagnostic Accuracy Studies) (230, 231); and STREGA initiative (STrengthening the REporting of Genetic Association Studies) (232, 233).

## Discussion

A methodological framework to design new experiments to study the effects of musical structures and noise on ECG and HRV signals was presented. This framework has three main components—experimental design and procedure, data analysis, and report of outcomes—and is able to be generalised to research related to other types of sound. Its objective is to provide guidelines to standardise new studies and thus facilitate comparison between study outcomes. AI techniques were considered as the main analysis tool as AI has recently revealed its advantages in studying different types of data, showing great capacity to deal with big data at a single stroke with efficiency, accuracy, and precision in analysis. Moreover, AI allows extracting features from complex, high-dimensional and noisy data, providing a high capability of generalisation and robustness.

Considering that new trends in AI suggest developing new metrics for each specific application, a cost matrix was introduced as an evaluation metric. Additionally, validation of the framework is presented, for which a clinical validation was adapted. As future work, this is a new proposal that needs to be validated in future experiments; moreover, it requires a series of studies with different types of patients and different sound techniques in order to be improved. This framework was developed to contribute to quality improvement of research associated with sound and music. This version constitutes an initial perusal of the perception field; it is expected that researchers can contribute with their own vision and experience to develop new, enhanced versions of the present framework.

## Data Availability Statement

The original contributions presented in the study are included in the article/supplementary material, further inquiries can be directed to the corresponding author/s.

## Author Contributions

EI-Á: conceptualisation, methodology, literature review, writing—original draft, and writing—review and editing. HL-C and FM-B: supervision and reviewing. LN: reviewing and editing. RV-C: conceptualisation, methodology, supervision, and reviewing and editing. All authors contributed to the article and approved the submitted version.

## Conflict of Interest

The authors declare that the research was conducted in the absence of any commercial or financial relationships that could be construed as a potential conflict of interest.

## Publisher's Note

All claims expressed in this article are solely those of the authors and do not necessarily represent those of their affiliated organizations, or those of the publisher, the editors and the reviewers. Any product that may be evaluated in this article, or claim that may be made by its manufacturer, is not guaranteed or endorsed by the publisher.

## Footnotes

^1^Available in http://ceur-ws.org/Vol-2747/paper6.pdf
